# Targeted Intracellular Metal Modulation Attenuates Oxidative Stress and Preserves Retinal Integrity and Function in a Zebrafish Model of Mitochondrial Dysfunction and Age-Related Retinal Degeneration

**DOI:** 10.3390/ijms27188265

**Published:** 2026-09-16

**Authors:** Itzchak Angel, Kalaichitra Periyasamy, Micha Gladnikoff, Benin Joseph, Raphael Mayer, Erez Aminov

**Affiliations:** 1 Telomir Pharmaceuticals, Inc., Miami, FL 33606, USA; erez@telomirpharma.com; 2 Pentagrit Discovery Laboratory, Chennai, Tamil Nadu 600100, India; kal@pentagrit.com (K.P.); ben@pentagrit.com (B.J.); 3 Smart Assays Biotechnologies, Nes Ziona 7414003, Israel; micha@assays.co.il (M.G.); mayer@assays.co.il (R.M.)

**Keywords:** retinal degeneration, age-related macular degeneration, JmjC histone demethylases, KDM inhibition, epigenetic reprogramming, intracellular metal modulation, oxidative stress, zebrafish model

## Abstract

Retinal degeneration is associated with mitochondrial dysfunction, oxidative stress, and disruption of metal homeostasis within the retinal pigment epithelium (RPE). Dysregulation of redox-active metals has been implicated in age-related macular degeneration (AMD), but whether targeted intracellular metal modulation preserves retinal structure and function remains incompletely understood. This study explores the effect of metal ion modulation on retinal structure and visual function. Telomir-Zn, a Zinc-based intracellular metal modulator, depletes labile Fe^2+^, potently inhibits multiple JmjC KDMs, and induces epigenetic reprogramming. In a zebrafish model combining impaired DNA repair with accelerated mitochondrial oxidative stress and progressive retinal degeneration (Sen57^wrn−/−^ND6^−/+^), Telomir-Zn reduced oxidative stress, preserved retinal architecture, improved visual behaviors, and partially normalized telomeric content and CpG methylation patterns. Complementary studies in human ARPE-19 RPE cells showed attenuation of iron/copper-induced ROS and calcium dysregulation, with reciprocal zinc accumulation and labile Fe^2+^ depletion. These findings suggest that targeted metal modulation and multi-KDM inhibition may be a novel strategy to mitigate oxidative-epigenetic stress to preserve retinal structure/function in degenerative disease, supporting further investigation and development for AMD.

## 1. Introduction

Retinal degeneration is characterized by mitochondrial dysfunction, oxidative stress, and impaired cellular homeostasis, particularly within the RPE—key features of AMD. Dysregulation of redox-active metals (iron and copper) promotes reactive oxygen species (ROS) generation via Fenton chemistry and contributes to lipid peroxidation, inflammation, and photoreceptor loss.

The retina is uniquely vulnerable to oxidative injury because of its high metabolic demand, oxygen consumption, and lipid-rich environment. Increasing evidence implicates dysregulation of redox-active transition metals, particularly iron and copper, as contributors to oxidative damage in the retina and RPE. Excess labile iron has been associated with lipid peroxidation, mitochondrial dysfunction, inflammatory signaling, and photoreceptor loss in experimental models and human AMD [1,2,3,4].

Iron and copper are essential for retinal physiology, serving critical roles in electron transport and enzymatic activity. However, when present in excess or improperly compartmentalized, these metals promote ROS generation through redox cycling and Fenton chemistry [5,6]. Oxidative stress and mitochondrial dysfunction influence chromatin regulation and genomic stability in retinal cells. Fe^2+^-dependent Jumonji C (JmjC) histone demethylases (KDM2, KDM5, KDM6 families) serve as critical sensors of intracellular metal and redox status. These enzymes regulate chromatin accessibility and expression of genes involved in inflammation, senescence, DNA repair, and cell survival [7,8,9,10]. Dysregulated KDM activity links metal imbalance to epigenetic alterations that exacerbate retinal degeneration. Zinc, by contrast, acts as a redox-inert structural cofactor for transcription factors and repair proteins.

Zinc represents a functionally distinct metal in this context. Unlike iron and copper, zinc is redox-inert under physiological conditions yet serves as an essential structural cofactor for numerous transcription factors, DNA repair proteins, and chromatin-associated complexes [11,12,13]. Intracellular zinc homeostasis is tightly regulated and intersects with iron metabolism through shared buffering and transport mechanisms. Disruption of zinc–iron balance has been associated with increased oxidative stress and impaired genomic stability in aging tissues, but its contribution to retinal degeneration remains incompletely defined.

The zebrafish (Danio rerio) provides a vertebrate system well suited for investigating retinal oxidative pathology because of its conserved retinal architecture, optical accessibility, and established models of retinal degeneration [14,15,16,17]. Zebrafish lines combining mitochondrial dysfunction and impaired DNA repair exhibit accelerated retinal degeneration characterized by photoreceptor loss, RPE disruption, elevated ROS, and visual impairment [18,19,20], recapitulating key features of oxidative retinal disease.

Telomir-Zn modulates intracellular metal pools (preferential Cu^2+^ > Fe^2+^ affinity), depletes labile Fe^2+^, and potently inhibits multiple JmjC KDMs [21]. This study evaluates whether such modulation preserves retinal integrity in a zebrafish model of accelerated oxidative retinal degeneration while providing mechanistic insights into metal–KDM–epigenetic axes.

Using Telomir-Zn, we evaluated effects on retinal architecture, visual behavior, oxidative stress, and genomic regulatory measures. Complementary experiments in human RPE cells examined whether intracellular metal redistribution alters redox-active metal pools and attenuates iron- and copper-induced oxidative stress. Together, these studies evaluate whether targeted intracellular metal regulation can stabilize redox balance and preserve retinal integrity in vivo.

## 2. Results

### 2.1. Metal-Dependent Modulation Attenuates Oxidative Stress and Calcium Dysregulation in Human Retinal Cells

Oxidative stress disrupts intracellular calcium homeostasis through release of calcium from mitochondria and other intracellular stores. To determine whether intracellular metal modulation influences metal-induced calcium dysregulation, ARPE-19 retinal pigment epithelial cells were challenged with Fe^2+^ or Cu^2+^, and intracellular calcium flux was quantified using the calcium-sensitive fluorescent probe Fluo-8.

**Figure 1:**
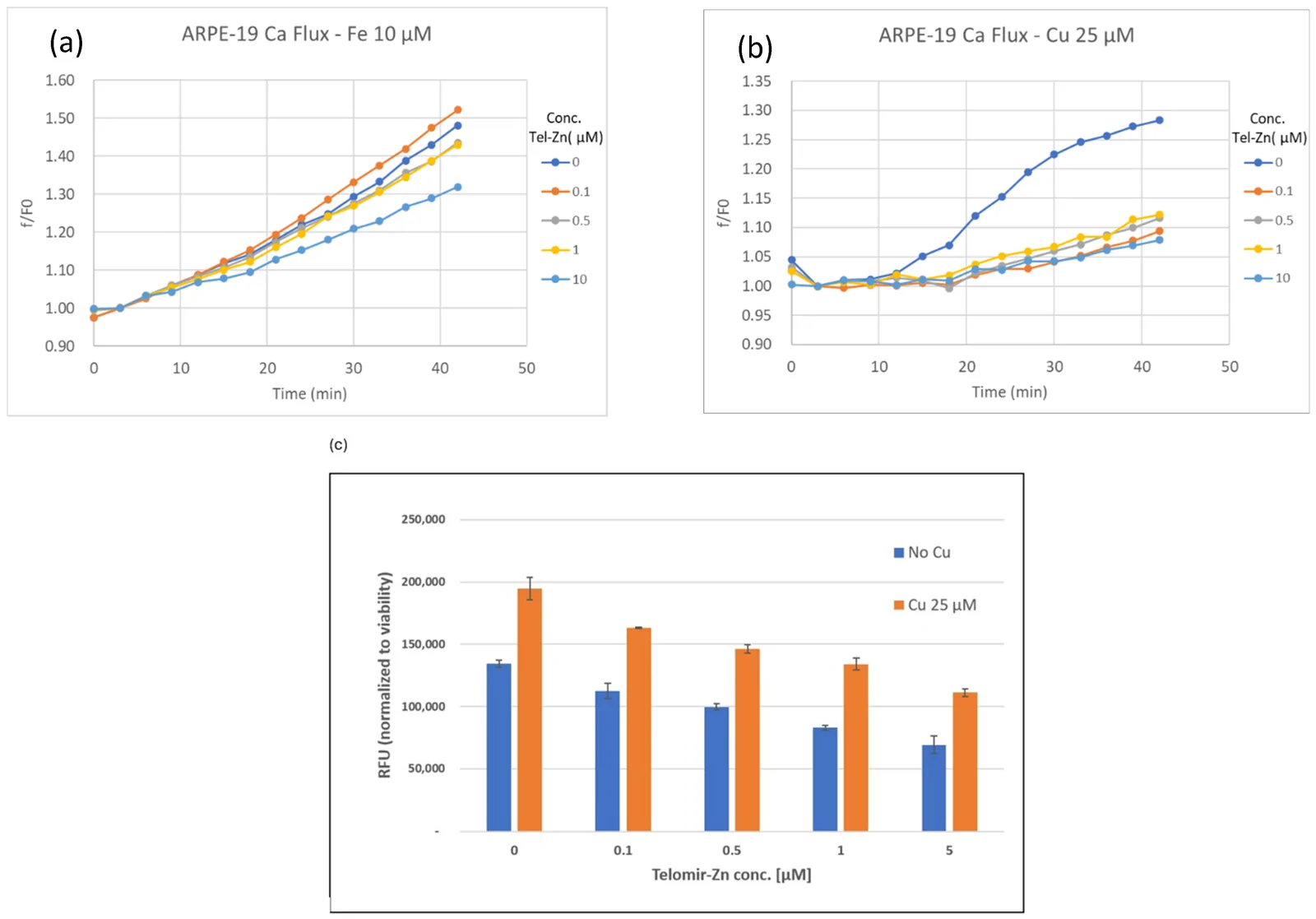
Metal-dependent modulation attenuates calcium dysregulation and oxidative stress in ARPE-19 cells. (**a**) Intracellular calcium flux in ARPE-19 cells following exposure to Fe^2+^ (10 µM) in the presence or absence of Telomir-Zn (0.1–5 µM). (**b**) Intracellular calcium flux following Cu^2+^ (25 µM) exposure with or without Telomir-Zn. Representative graphs are shown. Independent biological replicates (n = 3). (**c**) Intracellular reactive oxygen species (ROS) levels measured using 2′,7′-dichlorofluorescein diacetate (DCFH-DA) fluorescence following copper exposure. Data are presented as mean ± standard deviation (SD). Independent biological replicates (n = 3).

Exposure to Fe^2+^ (10 µM) induced a pronounced increase in intracellular calcium flux. Treatment with Telomir-Zn produced a dose-dependent attenuation of this response (Figure 1a). Similarly, Cu^2+^ challenge (25 µM) triggered a delayed but sustained elevation in intracellular calcium levels between 10 and 20 min, which was markedly reduced in the presence of Telomir-Zn across tested concentrations (Figure 1b).

Intracellular ROS levels were quantified using DCFH-DA fluorescence. Copper exposure resulted in a robust increase in ROS. Telomir-Zn reduced baseline oxidative stress and dose-dependently reversed copper-induced ROS accumulation, restoring ROS levels toward baseline at concentrations as low as 1 µM (Figure 1c).

### 2.2. Reciprocal Intracellular Zinc Accumulation and Labile Iron Depletion

To determine whether modulation of intracellular metal homeostasis alters labile metal pools, intracellular zinc and ferrous iron levels were quantified in a model cellular system, using cultured human HaCaT cells with complementary fluorescent probes. Labile zinc was measured using DA-ZP1, and labile ferrous iron (Fe^2+^) was assessed using FerroOrange.

**Figure 2:**
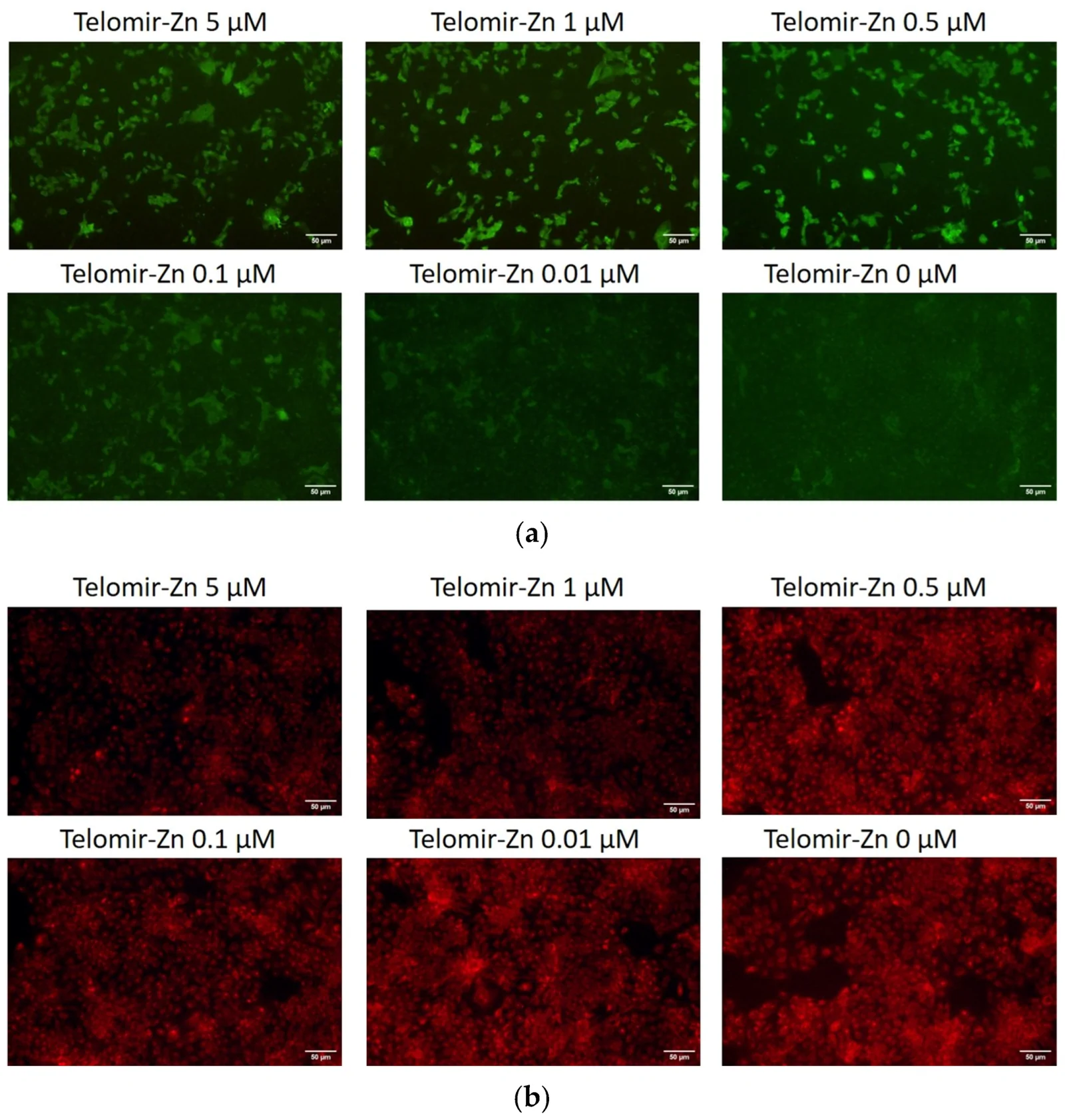
Reciprocal intracellular zinc accumulation and labile iron depletion following metal modulation (**a**) Representative fluorescence microscopy images of HaCaT cells stained with the zinc-selective probe DA-ZP1 following 2 h incubation with increasing concentrations of Telomir-Zn (0.01–5 µM). (**b**) Representative fluorescence images of FerroOrange staining demonstrating intracellular labile ferrous iron (Fe^2+^) levels following 2 h Telomir-Zn exposure. Images were acquired with an ICX41 fluorescence microscope at 10× magnification. Quantification was performed using biologically independent samples. Scale bar: 50 µm.

Short-term exposure to Telomir-Zn induced a rapid, dose-dependent increase in intracellular zinc fluorescence, detectable within 30 min and sustained for at least 2 h (Figure 2a). Zinc accumulation was observed at concentrations as low as 0.1–1 µM without detectable effects on cell confluence or viability.

In parallel, FerroOrange staining demonstrated a reciprocal decrease in intracellular labile Fe^2+^ levels across the same concentration range (Figure 2b). Increasing Telomir-Zn concentrations were associated with progressive suppression of FerroOrange fluorescence.

### 2.3. Telomir-Zn Potently Inhibits JmjC Histone Demethylases

Biochemical assays demonstrated low-nanomolar potency of Telomir-Zn against key KDM family members (Table 1). The strongest inhibition was observed for KDM5B (63 nM), followed by KDM2A (240 nM), KDM5A (310 nM), KDM2B (300 nM), KDM6A (390 nM), KDM5C (470 nM), and KDM6B (2000 nM). This multi-KDM blockade is expected to promote accumulation of activating histone marks and reduce aberrant DNA methylation at stress-response loci (Table 1).

### 2.4. Zebrafish Studies

#### 2.4.1. Improvement in Visual Performance

**Figure 3:**
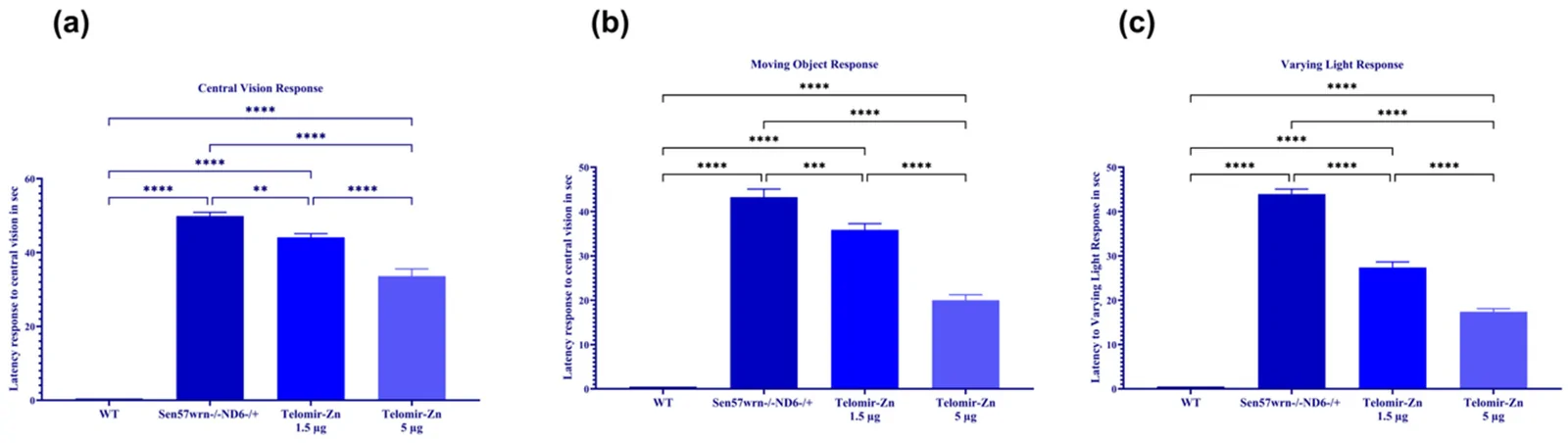
Metal-dependent modulation improves visual response latency in Sen57^wrn−/−^ND6^−/+^ zebrafish. (**a**) Latency to central visual response. (**b**) Latency to moving object tracking response. (**c**) Latency to variable light adaptation response. Data are presented as mean ± SD (n = 24 per group). Statistical analysis was performed using one-way ANOVA followed by Tukey’s multiple-comparisons post hoc test.

Sen57^wrn−/−^ND6^−/+^ zebrafish exhibited prolonged central visual response latency compared with WT controls (49.9 ± 4.9 s versus 0.5 ± 0 s; *p* < 0.0001). Telomir-Zn reduced latency in a dose-dependent manner (Figure 3a).

The degeneration model also demonstrated delayed moving object response and light adaptation latencies relative to WT animals ** *p* < 0.01, *** *p* < 0.001, **** *p* < 0.0001. Treatment significantly reduced response latencies at both doses, with greater improvement observed at the higher dose (Figure 3b,c).

#### 2.4.2. Retinal Structural Preservation

Histological examination revealed intact laminar retinal organization in wild-type zebrafish, whereas Sen57^wrn−/−^ND6^−/+^ animals exhibited pronounced retinal degeneration characterized by photoreceptor loss, thinning of the outer nuclear layer (ONL), disruption of the RPE, and disorganization of inner retinal layers.

**Figure 4:**
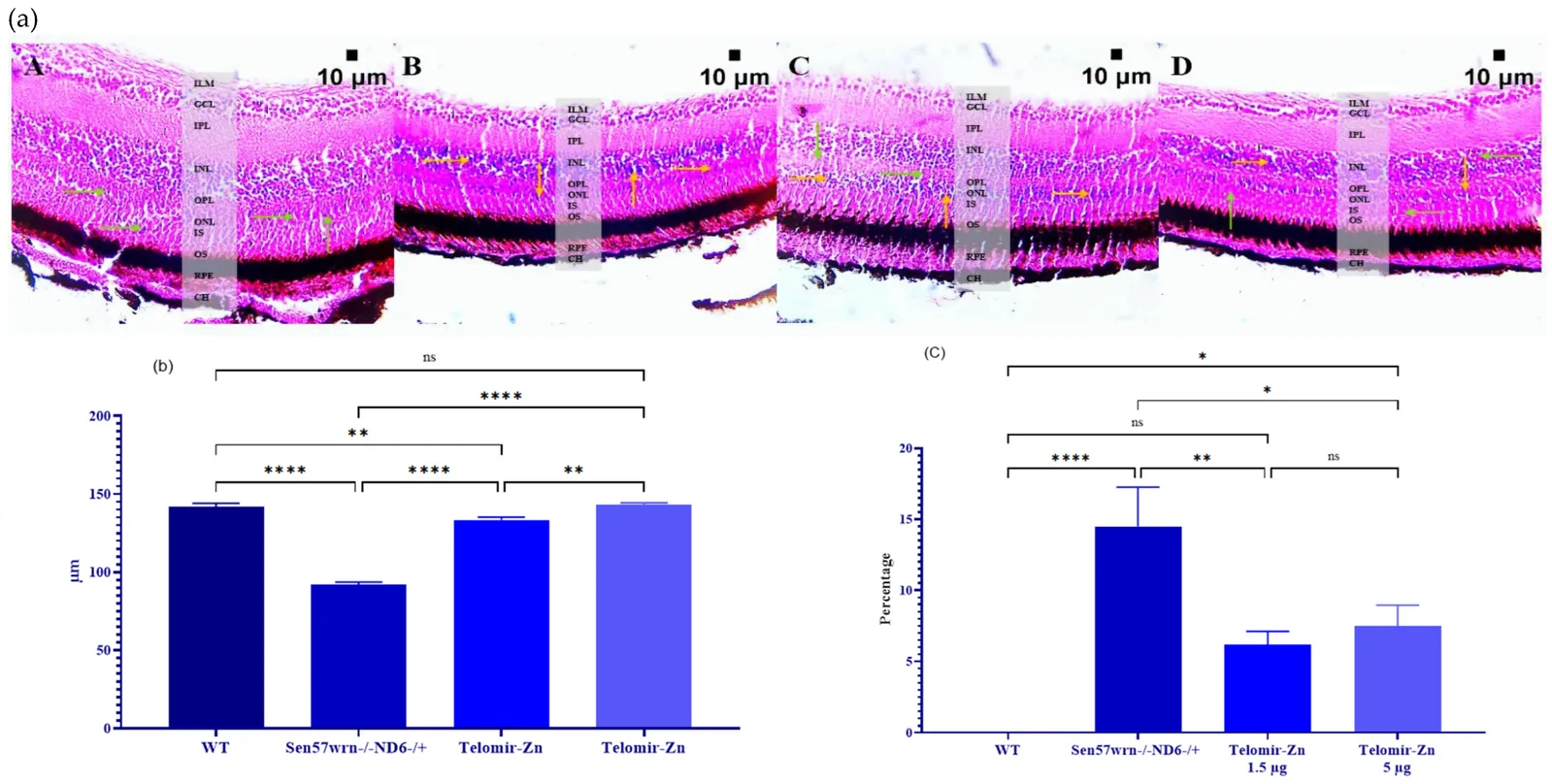
Retinal structural alterations and histological quantification. (**a**) Representative hematoxylin and eosin–stained cross-sections of adult zebrafish retina from wild-type (WT) A, Sen57^wrn−/−^ND6^−/+^, B and treated groups (1.5 µg, C and 5 µg, D). Retinal layers are labeled: retinal pigment epithelium (RPE), inner segment (IS), outer segment (OS), photoreceptor layer (PR), outer nuclear layer (ONL), outer plexiform layer (OPL), inner nuclear layer (INL), ganglion cell layer (GCL), and nerve fiber layer (NFL). Green arrows—healthy cells, yellow arrows—degenerated cells. (**b**) Quantification of overall retinal thickness. (**c**) Percentage of degenerated retinal cells. Data are presented as mean ± SD (n = 6 per group). Statistical analysis was performed using one-way ANOVA followed by Tukey’s post hoc test. * *p* < 0.05, ** *p* < 0.01, **** *p* < 0.0001. ns, non specific.

Quantitative analysis demonstrated a significant increase in retinal degeneration in the accelerated aging model compared with wild-type controls (14.5 ± 6.7% versus 0%; *p* < 0.001). Treated animals showed restoration of ONL, INL, OPL, and GCL thickness, indicative of structural stabilization and regenerative remodeling of retinal layers. Modulation of intracellular metal homeostasis significantly reduced the extent of retinal degeneration. Both doses produced a similar, significant reduction in degenerative burden (*p* < 0.05 versus model), indicating substantial stabilization of retinal architecture (Figure 4).

#### 2.4.3. Reduction of Mitochondrial Oxidative Stress in an Accelerated Retinal Degeneration Model

Brain ROS levels were quantified as a surrogate measure of mitochondrial oxidative stress, as technical variability limited reliable whole-retina ROS measurements in adult fish [20]. Brain ROS levels were significantly elevated in Sen57^wrn−/−^ND6^−/+^ zebrafish compared with wild-type (WT) controls (3.65 ± 0.44-fold increase; *p* < 0.0001), consistent with mitochondrial dysfunction.

**Figure 5:**
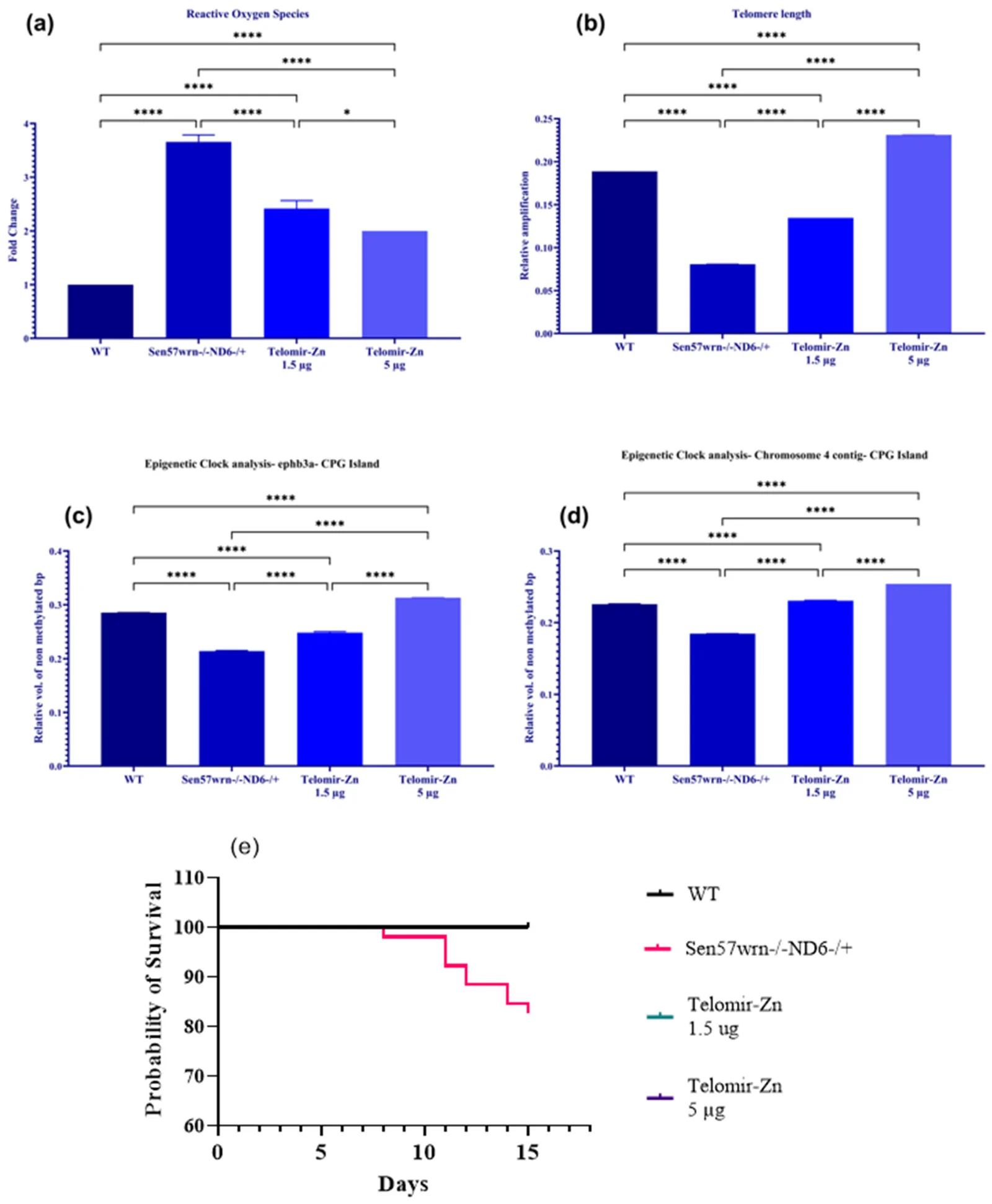
Metal-dependent modulation reduces oxidative stress and alters genomic measures. (**a**) Brain reactive oxygen species (ROS) levels measured in wild-type (WT), Sen57^wrn−/−^ND6^−/+^, and treated zebrafish. (**b**) Relative telomeric DNA content assessed by PCR-based comparative amplification. (**c**) Relative non-methylated cytosine–phosphate–guanine (CpG) volume at aging-associated loci. (**d**) DNA methylation analysis of chromosome 4 CpG island regions. (**e**) Kaplan–Meier survival curves across experimental groups (n = 52 per group). Data are presented as mean ± SD. Molecular analyses were performed using one-way ANOVA followed by Tukey’s post hoc test (n = 12 per group unless otherwise specified). Survival analysis was conducted using the log-rank (Mantel–Cox) test. * *p* < 0.05, **** *p* < 0.0001.

Modulation of intracellular metal homeostasis significantly reduced brain ROS levels at both tested doses, with the higher dose restoring ROS values toward WT baseline (*p* < 0.0001 versus model; Figure 5a).

#### 2.4.4. Epigenetic and Telomere-Associated Measures

Relative telomeric DNA content was significantly reduced in Sen57^wrn−/−^ND6^−/+^ zebrafish compared with WT controls (*p* < 0.0001). Telomir-Zn treatment was associated with a dose-dependent increase in relative telomeric DNA content (Figure 5b).

DNA methylation analysis at aging-associated cytosine–phosphate–guanine (CpG) loci demonstrated significant alterations in the accelerated degeneration model (*p* < 0.0001). Treatment was associated with partial restoration of methylation patterns at ephb3a and chromosome 4 CpG island regions (Figure 5c,d).

Kaplan–Meier analysis revealed reduced survival in Sen57^wrn−/−^ND6^−/+^ zebrafish (83%) compared with WT controls (100%). Both treated groups exhibited improved survival over the study period (Figure 5e).

#### 2.4.5. Systemic Measures: Body Weight and Muscle Density

**Figure 6:**
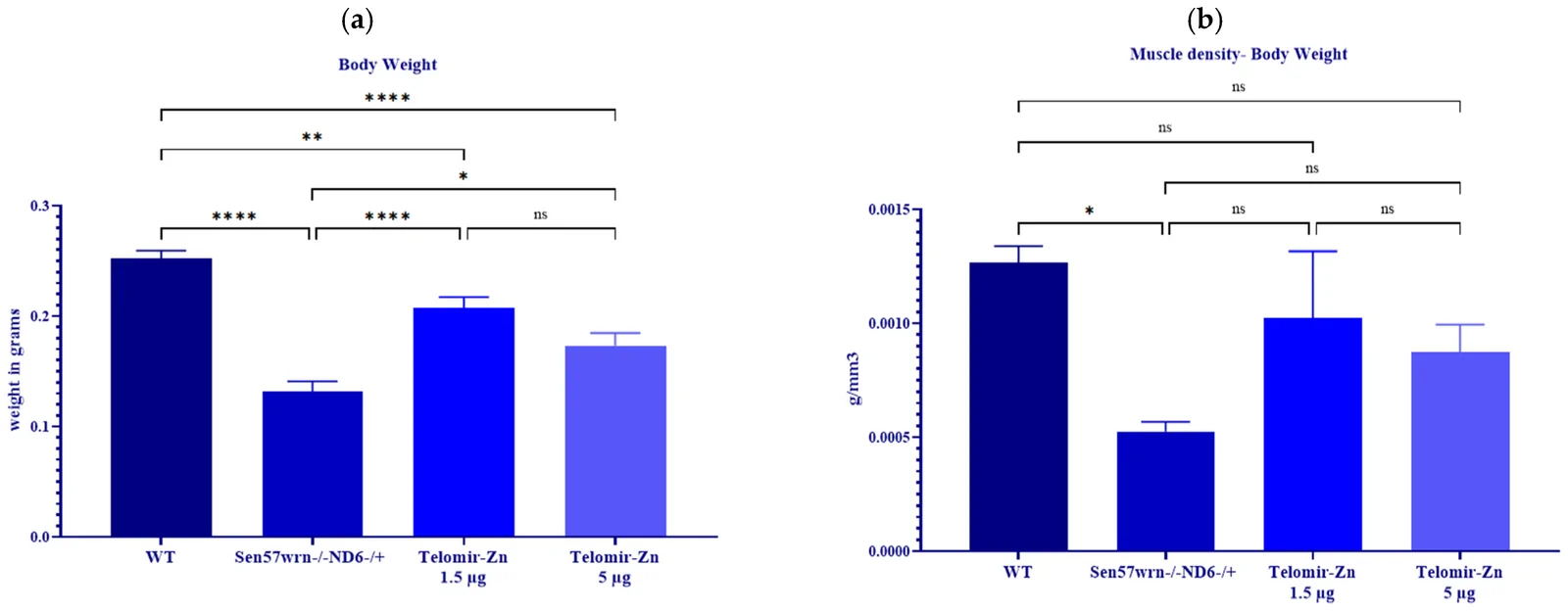
Body weight and muscle density measurements. (**a**) Body weight of adult zebrafish across experimental groups. (**b**) Whole-body density measurements as an indirect indicator of muscle mass. Data are presented as mean ± SD (n = 12 per group). Statistical analysis was performed using one-way ANOVA followed by Tukey’s post hoc test. * *p* < 0.05, ** *p* < 0.01, **** *p* < 0.0001. ns, non specific.

Sen57^wrn−/−^ND6^−/+^ zebrafish exhibited reduced body weight and muscle density compared with WT controls. Treatment was associated with increased body weight and improved muscle density relative to the untreated model (Figure 6).

#### 2.4.6. Blastema Progenitor Cell Volume

**Figure 7:**
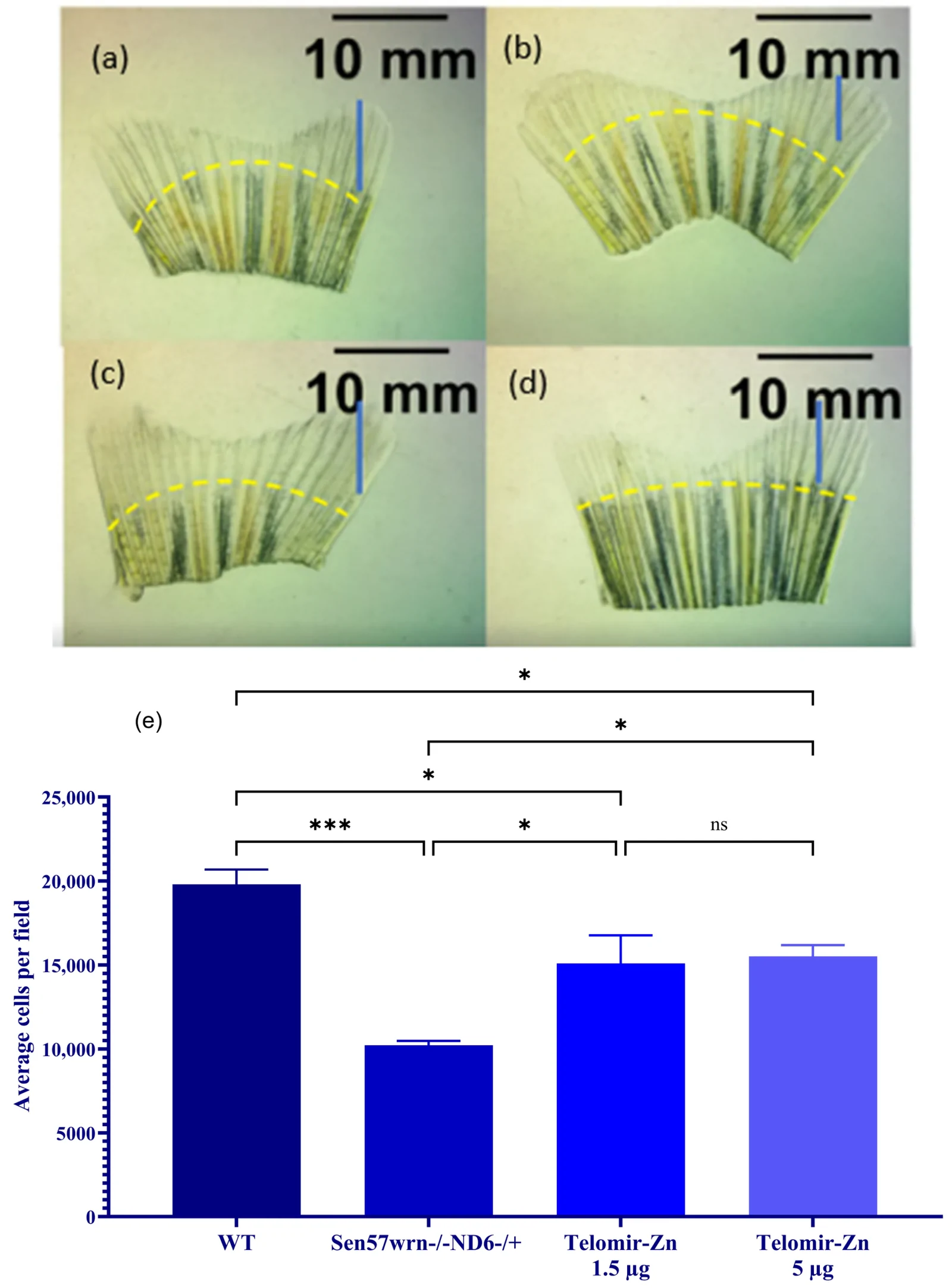
Blastema progenitor cell volume following caudal fin regeneration. (**a**–**d**) Representative stereomicroscopic images of adult zebrafish caudal fins following amputation and regeneration (1× magnification). The blue line denotes the regenerated caudal fin region post-amputation. The yellow dashed line marks the original amputation site. (**e**) Quantification of blastema progenitor cell volume across experimental groups. Data are presented as mean ± SD (n = 4–6 per group). Statistical analysis was performed using one-way ANOVA followed by Tukey’s post hoc test. * *p* < 0.05, *** *p* < 0.001. ns, non specific.

Sen57^wrn−/−^ND6^−/+^ zebrafish exhibited reduced blastema progenitor cell volume following caudal fin amputation compared with WT animals (*p* < 0.001). Treatment increased blastema progenitor cell volume at both tested doses (Figure 7).

## 3. Discussion

This study demonstrates that modulation of intracellular metal homeostasis reduced oxidative stress and preserved retinal structure and visual performance in a zebrafish model of accelerated retinal degeneration. Across behavioral, histological, molecular, and cellular endpoints, treatment was associated with improved visual response latency and attenuation of retinal structural disruption.

Iron and copper dysregulation have been increasingly implicated in AMD and other retinal degenerative conditions. Excess labile iron can amplify oxidative injury through Fenton chemistry, promoting lipid peroxidation, mitochondrial dysfunction, and RPE damage. The present findings support the concept that modulation of intracellular redox-active metal balance may represent a complementary strategy for limiting oxidative stress in degenerative retinal disease.

In the accelerated degeneration zebrafish model, ND6 deficiency was associated with elevated brain ROS levels, consistent with mitochondrial dysfunction. Treatment reduced ROS levels toward baseline, suggesting stabilization of redox homeostasis. Although retinal ROS measurements were technically limited in adult animals, complementary cellular studies in human RPE cells demonstrated attenuation of copper- and iron-induced oxidative stress and calcium dysregulation. These findings are consistent with intracellular redistribution of redox-active metal pools rather than simple extracellular chelation.

The observed reciprocal increase in intracellular zinc and reduction in labile ferrous iron suggest that modulation shifts intracellular metal availability toward a less redox-reactive state. Zinc is redox-inert under physiological conditions and serves structural and regulatory roles in numerous proteins. Increasing intracellular zinc while reducing labile Fe^2+^ may decrease susceptibility to oxidative stress without broadly disrupting essential metal-dependent processes.

Improvements in visual response latency were accompanied by reduced structural disruption of the outer nuclear layer and photoreceptor compartments. The concordance between behavioral and histological findings supports the possibility that metal-dependent redox modulation influences retinal tissue integrity under conditions of mitochondrial stress.

Alterations in DNA methylation patterns and relative telomeric DNA content were observed in the accelerated degeneration model and were modified following treatment. Although the functional implications of these genomic measures require further investigation, iron-dependent chromatin-modifying enzymes are sensitive to intracellular redox state and metal availability. In a related study, using Telomir-Zn in a mouse xenograft model of prostate cancer [21], it was shown that Telomir-Zn reduced promoter DNA-methylation in several tumor-suppressor-associated genes, including CDKN2A, GSTP1, RASSF1A, MASPIN, CASP8, STAT1, and TMS1. These findings suggest that modulation of redox-active metals may influence broader cellular stress-response pathways through effects on DNA methylation.

In the current study, systemic measures, including body weight, muscle density, and blastema progenitor cell volume, were also modified following treatment. These findings should be interpreted cautiously, and further studies will be necessary to determine tissue specificity and relevance to retinal disease.

Several limitations of the present study should be acknowledged. First, the treatment duration was relatively short (14 days). While this timeframe was sufficient to demonstrate rapid improvements in visual function, retinal structural preservation, and reduction of oxidative stress, longer-term studies will be necessary to evaluate the durability of these protective effects and potential disease-modifying benefits over extended periods of degeneration.

Second, due to technical constraints associated with reliable ROS measurement in the small retinal tissue of adult zebrafish, we used brain ROS levels as a surrogate marker of mitochondrial oxidative stress. However, the complementary in vitro data in human ARPE-19 retinal pigment epithelial cells, which demonstrated dose-dependent attenuation of both iron- and copper-induced ROS production and calcium dysregulation, strongly support the translational relevance of reduced oxidative burden in retinal cells.

Third, although the Sen57^wrn−/−^ND6^−/+^ zebrafish line effectively recapitulates key pathological features of oxidative stress, mitochondrial dysfunction, and accelerated retinal degeneration relevant to human AMD, it does not fully model the complex, chronic, and multifactorial nature of age-related macular degeneration in humans. Additional validation in mammalian models of retinal degeneration (e.g., iron-overload or photo-oxidative damage models) will be important to further establish therapeutic potential.

Also, as the iron and zinc intracellular measurements were not carried out in RPE-cells, it remains to be verified that the same process is also manifested in these cell lines.

The potent multi-KDM inhibitory profile of Telomir-Zn provides a potential mechanistic link between intracellular Fe^2+^ depletion and the observed reductions in oxidative stress, partial normalization of CpG methylation and telomeric content, and preservation of retinal structure and function. By inhibiting Fe^2+^-dependent demethylases such as KDM5B and KDM6B—implicated in senescence, inflammation, and oncogenic and stress-response programs—Telomir-Zn may promote a more protective epigenetic landscape in RPE cells and photoreceptors under oxidative stress [24]. Further in vitro and in vivo studies are planned to investigate KDM target engagement, relevant histone methylation marks, and KDM-dependent transcriptional changes, thereby directly evaluating the link between KDM inhibition and retinal effects.

These findings extend the therapeutic paradigm of metal modulation from oncology (where KDM inhibition suppresses tumor growth via epigenetic reprogramming [21]) to retinal degeneration, highlighting a conserved vulnerability at the intersection of metal homeostasis, redox balance, and chromatin regulation. While we demonstrate metal modulation in both keratinocytes and RPE cells, the functional consequences for retinal degeneration require validation in the retinal context.

Finally, while treatment was associated with partial normalization of relative telomeric DNA content and selected CpG methylation patterns, the functional consequences of these epigenetic changes on photoreceptor survival and RPE integrity remain to be fully elucidated. Future mechanistic studies will be required to establish causal relationships between intracellular metal redistribution, epigenetic regulation, and long-term retinal protection.

The absence of a WT + Telomir-Zn group limits interpretation of Telomir-Zn’s effects in healthy animals and should be addressed in future studies. Telomir-Zn improved systemic measures, including muscle mass, body weight, and ROS levels. These effects may have contributed to the observed improvements in visual responses and retinal structure. Inflammatory parameters that may mediate these effects also require investigation.

Despite these limitations, the convergence of behavioral, histological, cellular, and molecular findings provides promising preliminary evidence that modulation of intracellular metal homeostasis may mitigate oxidative retinal damage. Future studies should evaluate these effects in additional AMD-specific models, such as aged mice, complement-driven inflammation models, and iPS cells derived from patients with AMD.

In summary, modulation of intracellular redox-active metal balance was associated with reduced oxidative stress and preservation of retinal structure and visual performance in a vertebrate model of retinal degeneration. These findings are consistent with a model in which intracellular metal redistribution stabilizes mitochondrial redox homeostasis and may influence genomic regulatory pathways that contribute to retinal integrity.

## 4. Materials and Methods

### 4.1. Telomir-Zn

Telomir-Zn is a zinc-complexed small molecule consisting of 2,4,6-tris(3,4-dihydro-2H-pyrrol-2-yl)pyridine coordinated with ZnCl_2_. The compound was synthesized by Recipharm (Now Sinai Immunotherapeutics, Yavne, Israel) and supplied at a purity of 95%.

### 4.2. Cell Culture

ARPE-19 human retinal pigment epithelial cells (ATCC CRL-2302, Manassas, VA, USA) and HaCaT human keratinocytes (AddexBio T0020001, San Diego, CA, USA) were cultured in Dulbecco’s Modified Eagle Medium/Ham’s F-12 (DMEM/F12; Biowest, L0093, Riverside, MO, USA) and DMEM (Biowest, L0102), respectively. Media were supplemented with 10% fetal bovine serum (FBS; Gibco, A5256701, Waltham, MA, USA) and 1% penicillin–streptomycin (Gibco, 15140-122, Grand Island, NY, USA). HaCaT cultures were additionally supplemented with 2 mM sodium pyruvate (Sartorius, 03-042-1B, Göttingen, Germany).

Cells were maintained at 37 °C in a humidified incubator with 5% CO_2_. ARPE-19 and HaCaT cells were seeded into 96-well plates at densities of 20,000 and 10,000 cells per well, respectively. Experiments were performed 2–5 days after seeding at approximately 90% confluence.

### 4.3. Reactive Oxygen Species (ROS) Assay

Cells were incubated with Telomir-Zn at final concentrations of 0.1, 0.5, 1, or 5 µM in complete culture medium, with or without copper chloride (CuCl_2_; 25 µM; Sigma, C3279, St. Louis, MO, USA), for 24 h at 37 °C.

Following incubation, cells were washed twice with Hanks’ Balanced Salt Solution (HBSS) and stained with 2′,7′-dichlorofluorescein diacetate (DCFH-DA, Sigma, St. Louis, MO, USA) for 2 h at 37 °C. Fluorescence was measured using a microplate reader (CLARIOstar, BMG Labtech, Ortenberg, Germany).

Cell viability was assessed in parallel using the WST-1 assay (Sigma-Aldrich, 5015944001, St. Louis, MO, USA) according to the manufacturer’s instructions. Reactive oxygen species (ROS) values were normalized to cell viability.

All experiments were performed with three independent biological replicates.

### 4.4. Calcium Flux Assay

Cells were incubated with Telomir-Zn at final concentrations of 0.1, 0.5, 1, or 5 µM for 1 h at 37 °C. Cells were then washed and equilibrated in assay buffer consisting of HBSS supplemented with 0.1 mM calcium chloride (CaCl_2_), 0.9 mM magnesium chloride (MgCl_2_), and 20 mM HEPES.

Cells were loaded with the calcium-sensitive fluorescent dye Fluo-8 (AAT Bioquest, 21081, Pleasanton, California, USA) in the presence of probenecid (Sigma, P8761) for 1 h at 37 °C. Telomir-Zn was maintained at designated concentrations during dye loading.

Following dye loading, cells were challenged with Cu^2+^ (25 µM) or Fe^2+^ (10 µM). Fluorescence intensity was recorded at 2 min intervals for 40 min using a microplate reader.

All experiments were performed with three independent biological replicates. No statistical analysis was carried out in these experiments.

### 4.5. Intracellular Zinc and Iron Fluorescence Imaging

HaCaT cells were seeded into 96-well plates and maintained as described above.

For zinc detection, cells were incubated with Telomir-Zn (0.01–5 µM) for 2 h at 37 °C and stained with DA-ZP1 (1 µM; R&D Systems, 7444/2) for 30 min. Cells were washed twice with HBSS prior to imaging.

For labile ferrous iron detection, cells were incubated with Telomir-Zn (0.01–5 µM) for 2 h and stained with FerroOrange (1 µM; Dojindo, F374, Mashiki-machi, Kumamoto Prefecture, Japan) for 30 min at 37 °C.

Fluorescence imaging was performed using a fluorescence microscope (ICX41, Ningbo Sunny Instruments Co., Ltd., Yuyao City, Ningbo, China) at 10× magnification. DA-ZP1 was imaged with excitation at 480 nm and emission collected using a 510–550 nm band-pass filter. FerroOrange was imaged with excitation at 545 nm and emission collected using a 575–650 nm band-pass filter.

### 4.6. Histone Demethylase (KDM) Assay

The inhibitory activity of Telomir-Zn against recombinant JmjC-domain histone demethylases was evaluated using established biochemical assays as previously described [22,23]. Briefly, Sf9 insect cells were used to express individual human recombinant KDM enzymes (KDM2, KDM5, and KDM6 family members). Enzyme activity was measured in the presence of varying concentrations of Telomir-Zn using biotinylated histone peptide substrates specific to each family: Biotin-H3K36me1 for KDM2 enzymes, Biotin-H3K4me2 for KDM5 enzymes, and Biotin-H3K27me2 for KDM6 enzymes. Demethylation reactions were quantified by detecting the release of formaldehyde or through antibody-based detection of remaining methyl marks. IC_50_ values were determined from dose–response curves performed in duplicate or triplicate.

### 4.7. Zebrafish Husbandry and Ethics

Wild-type and genetically modified zebrafish (*Danio rerio*) were bred and maintained at the Pentagrit Discovery facility under standard laboratory conditions (27 ± 1 °C; pH 7.2–7.4; 14 h light/10 h dark cycle). Fish were fed commercial flake food twice daily.

All experimental procedures adhered to the ARVO Statement for the Use of Animals in Ophthalmic and Vision Research. Studies were conducted at an AAALAC International–accredited facility and were reviewed and approved by the Institutional Animal Ethics Committee (IAEC). All protocols complied with the guidelines of the Committee for the Control and Supervision of Experiments on Animals (CCSEA), Government of India. Ethical approval number: PENT014 dated 19/07/2025.

### 4.8. Accelerated Retinal Degeneration Zebrafish Model

An accelerated degeneration zebrafish line (Sen57^wrn−/−^ND6^−/+^) was generated using N-ethyl-N-nitrosourea (ENU) mutagenesis followed by selective inbreeding. This model combines deficiency of Werner syndrome helicase (*wrn*) with mitochondrial electron transport chain dysfunction resulting from *ND6* mutation.

Heterozygous founders were intercrossed to generate mutants. Adult zebrafish were aged to 18 months prior to experimental intervention.

### 4.9. Experimental Groups and Treatment Administration

#### 4.9.1. Generation of Sen57^wrn−/−^ND6^−/+^ Line

WT-wrn^pgt63/pgt63(p.Leu960*) was generated by exposing WT F0 adult male zebrafish to ENU mutagenesis [25] that were bred to generate F1 offspring. Individual F1 larvae were screened for knockouts by PCR amplification of DNA from fin clips. Animals lacking the gene were analyzed by Sanger sequencing and the site was identified as a truncation at position 961 of the wrn gene. To generate stable nd6 mutants, adult female lines were continuously inbred for at least three generations and exposed to ENU. They were crossed with WT-wrn^pgt63/pgt63(p.Leu960*) adult males and larvae were selected by heightened sensitivity to hypoxia to identify double mutants. From the selected larvae, a linear amplification was performed by using two primers with shared forward primers: one at the N terminal and C terminal to identify mutation sites. Upon screening, heterogenous nd6 mutants were identified showing one allele with a truncation at site 76 nucleotide of the gene, and one truncated allele and one full-length allele. The WT-wrn^pgt63/pgt63(p.Leu960*); mt-nd6^pgt121/+ (p.Tyr25*) line was thus established and maintained under laboratory conditions as a separate colony. Adults from this colony were used for the study.

#### 4.9.2. Zebrafish Husbandry

All WRN mutant embryos were housed and maintained in embryo medium at the Pentagrit facility until use in the study. The mutants were continuously monitored at different developmental stages from embryonic to larval phases. Selected larvae were transferred to a two-liter housing container and subsequently housed at a density of 80 larvae per 25 L tank, ensuring sufficient swimming space and minimizing overcrowding.

During development from larvae to adults (0 dpf to 18 months), the fish were maintained under controlled conditions, with water temperature set at 27 ± 1 °C, light/dark cycle of 14/10hr and pH maintained between 7.2 and 7.4.

Adult zebrafish were allocated to experimental groups following genotypic confirmation. Each group consisted of 52 biologically independent animals. For block randomization, animals were first separated into smaller subgroups of three animals each before pooling multiple subgroups into a single larger group. Also, tanks and animal samples were coded with study codes and the technicians executing the study were unaware of group identities because they followed a coded system. This ensured the study was blinded.

Four groups were studied:

1. Wild-type (WT)
2. Sen57^wrn−/−^ND6^−/+^ mutants
3. Sen57^wrn−/−^ND6^−/+^ treated with Telomir-Zn (1.5 µg)
4. Sen57^wrn−/−^ND6^−/+^ treated with Telomir-Zn (5 µg)

#### 4.9.3. Dosing

Formulated Feed: Preparation of Study-Compound-Infused Feed and Feeding Cycle

To prepare the infused oral feeds, the required quantity of the test compound per day was mixed with standard commercial fish feed 4 mg and was mixed well until complete homogenization.

Telomir-Zn was administered individually at two dose levels of 1.5 and 5 µg/fish/day, corresponding to approximately 3 and 10 mg/kg/day, respectively, based on an average fish body weight of 0.5 g. Pellets were extruded and prepared at a dry weight of 4 mg per pellet for all the study groups. The study groups were fed one compound-infused pellet per fish every 24 h during the light cycle. During dosing, the fish were isolated from the study tanks and were fed individually to verify consumption. Observations on effects of the doses indicated systemic exposure. Vehicle fish were fed with commercial fish feed under conditions similar to those of the treated groups. The 1.5- and 5-µg doses correspond to individual daily doses for each animal.

#### 4.9.4. Washout

Housing water washout was performed once every 24 h to maintain homeostasis, minimize the accumulation of metabolic waste, and reduce the risk of mortality, thereby improving the overall survival and well-being of the zebrafish.

### 4.10. Visual and Behavioral Assessments

Visual function was evaluated using assays assessing central visual response, moving object tracking, and adaptation to changes in light intensity.

The Central Vision Response (CVR) assay is a behavioral paradigm used to assess central visual function in adult zebrafish. During the screening phase, 24 adult zebrafish (N = 24) were randomly selected from each experimental group for testing. The assay was conducted in a custom-designed test chamber equipped with a directional light source aimed to stimulate the central visual field. Prior to testing, all subjects were acclimatized to the experimental tank under controlled conditions without exposure to strong external stimuli, to minimize stress and habituation effects. The maximum observation period (cutoff time) for the assay was set at 5 min. The primary outcome measured was the latency to response—the time taken by the zebrafish to exhibit a behavioral reaction to the central light stimulus directed through the midline of the visual field. This parameter was recorded and used for subsequent quantitative analysis of central visual responsiveness.

The Moving Object Response (MOR) assay is a behavioral test designed to evaluate sensory, motor, and cognitive responses of adult zebrafish to a dynamic visual stimulus. During the screening phase, 24 adult zebrafish (N = 24) were randomly selected from each experimental group. The experimental apparatus consisted of a dual-chamber tank: a static inner chamber housing the test fish, and a concentric rotating outer drum that served as the source of the moving visual stimulus. The outer drum was patterned with markers simulating a moving object and was rotated at a constant speed of 20 revolutions per minute (RPM). Prior to stimulus exposure, the fish were allowed a 5 min acclimation period in the inner chamber under minimal external stimulation to reduce stress and baseline activity. Following acclimation, the rotating drum was activated, and the entire setup was continuously recorded using a video-tracking system (Canon, GDS3120B, Chennai, India). The primary endpoint measured was the latency to respond—defined as the time taken by the zebrafish to initiate a behavioral response to the moving object. This parameter was quantified and analyzed to assess sensory-motor integration and cognitive processing.

The Varying Light Response assay evaluates responses to changes in light color and intensity in adult zebrafish. During screening, 24 adult zebrafish were selected from each study group. The experimental setup consisted of a behavior-recording tank positioned over a light source that produced varying light intensities. The fish underwent a 5 min acclimation period without strong external stimuli. After acclimation, the fish were introduced into the center of the test tank, and light intensity was changed at random intervals. Response latency was recorded by video for subsequent analysis.

While no major differences in locomotion were observed between the groups, the potential locomotor/systemic effects of Telomir-Zn could influence visual-response latency was not directly evaluated.

### 4.11. Histological Analysis

Following 14 days of treatment, adult zebrafish (n = 6 per group) were euthanized, and eyes were dissected and fixed in 5% neutral buffered formalin for 48 h. Tissues were dehydrated, cleared, and embedded in paraffin.

Paraffin sections (10 µm) were stained with hematoxylin and eosin (H&E). Images were captured using light microscopy (Labomed LX-400, Haryana, India). Quantification of retinal degeneration was performed using QuPath software 0.6.0 in a masked manner. Degeneration percentage was calculated as degenerated cells divided by total cells × 100.

### 4.12. Reactive Oxygen Species Measurement in Zebrafish Brain

Brain ROS levels were quantified using a fluorometric assay (ROS Detection Kit; Sigma-Aldrich, MAK143). Brains (n = 12 per group) were isolated and homogenized in assay buffer. Total protein concentration was determined, and equal amounts of protein were used for each reaction.

Samples were incubated with detection reagent for 30 min at 37 °C. Fluorescence was measured at excitation/emission 490/525 nm. Values were normalized to total protein content.

### 4.13. DNA Methylation Analysis

Genomic DNA was isolated from fin tissue (n = 6 per group, pooled in pairs) using a silica column–based kit (QIAamp Mini Kit, Qiagen, Venlo, The Netherlands). DNA was subjected to sodium bisulfite conversion followed by locus-specific amplification.

Sodium bisulfite treatment

Genomic DNA was denatured by heating in a boiling water bath for 20 min. Freshly prepared 3 M NaOH (0.5 µL) and 5 M sodium bisulfite solution (20 µL) were added to the DNA. Each milliliter of the light-sensitive 5 M sodium bisulfite solution contained 0.475 g sodium bisulfite, 0.625 mL deionized water, 175 µL of 2 M NaOH, and 125 µL of 1 M hydroquinone. Heavy mineral oil (100 µL) was layered over 10 µL of DNA solution to minimize evaporation, and the solution was incubated in the dark at 50 °C for 12 h. After incubation, the mineral oil was removed. The following genomic sites were evaluated for CpG methylation: NP_571487.2, NP_956466.1, NP_956281.1, NP_001077335.1, and NP_001004550.1.

All the primers used in the study (Table 2) were designed using the NCBI Primer-BLAST version 2.17.0 tool. The primers were synthesized from Sigma-Aldrich (Bangalore, India) and the primers listed below were used in the study. After PCR amplification, the PCR products were quantified. A 1 µL aliquot of the sample was diluted in 100 µL of distilled water and analyzed by UV spectrophotometry (LMSP-UV 1200, LABMAN, Chennai, India) at 260 nm to determine the concentration of the DNA.

**Table 2:** Primer Sequences Used to Quantify DNA methylation by PCR.

Gene Mix	Primer Sequence Forward	Primer Sequence Reverse	Product Length
CHR2	CGGTGACATTCTGCATCCCA	AAACATTGGCATGTGCGGTT	109
CHR4	TTTCATCTGCAGTGACCACAT	GGAGATGGGGATTGTGATCCG	103

### 4.14. Relative Telomeric DNA Content

Relative telomeric DNA content was assessed using a PCR-based comparative amplification method. Genomic DNA was amplified in triplicate under identical reaction conditions. Amplification products were resolved on agarose gels and quantified by densitometric analysis. Relative telomeric content was calculated based on comparative amplification intensity across groups, using primers as descibed in Table 3 [26].

**Table 3:** Primer Sequences Used to Quantify relative Telomere Length by PCR.

Gene Mix	Primer Sequence Forward	Primer Sequence Reverse
Tel1b Tel21b	CGGTTTGTTTGGGTTTGGGTTTGGGTTTGGGTTTGGGTT	CAGCCGAAAGGCCCTTGGCAGGAGGGCTGCTGGTGGTCTACCCTT

### 4.15. Survival Analysis

Mortality was recorded daily throughout the 14-day treatment period. Survival curves were generated using the Kaplan–Meier method. Differences between groups were evaluated using the log-rank (Mantel–Cox) test. No animals were censored during the study period.

### 4.16. Statistical Analysis

Statistical analyses were performed using GraphPad Prism (version 10; GraphPad Software, San Diego, CA, USA). Data are presented as mean ± standard deviation (SD). Comparisons among multiple groups were performed using one-way analysis of variance (ANOVA) followed by Tukey’s multiple-comparisons post hoc test, unless otherwise specified. Survival curves were analyzed using the Kaplan–Meier method with group comparisons performed using the log-rank (Mantel–Cox) test.

All analyses were conducted using biologically independent samples. Sample sizes for each experiment are indicated in the corresponding figure legends. All tests were two-tailed, and a *p* value < 0.05 was considered statistically significant.

## 5. Conclusions

Modulation of intracellular redox-active metal balance was associated with reduced oxidative stress and preservation of retinal structure and visual performance in a zebrafish model of accelerated retinal degeneration. Complementary cellular studies demonstrated an iron-to-zinc exchange and attenuation of copper-induced oxidative stress and calcium dysregulation.

These findings support a role for metal-dependent regulation of mitochondrial redox homeostasis in retinal degenerative processes. Given the proposed contribution of oxidative stress and altered metal-ion handling to age-related macular degeneration, targeted modulation of intracellular metal balance merits further investigation in mammalian models of retinal disease.

## Figures and Tables

**Table 1:** Inhibitory Potency of Telomir-Zn Against JmjC Histone Demethylases (KDMs).

KDM Family	Specific Enzyme (Alias)	Primary Methylation Target(s)	Telomir-Zn Effect IC_50_, nM
KDM2	KDM2A (FBXL11)	H3K36me2	240
	KDM2B (FBXL10)		300
KDM6	KDM6A (UTX)	H3K27me3	390
	KDM6B (JMJD3)		2000
KDM5	KDM5A (JARID1A)	H3K4me3/H3K4me2	310
	KDM5B (JARID1B)		63
	KDM5C (JARID1C)		470

The inhibitory activity of Telomir-Zn against recombinant JmjC-domain histone demethylases was evaluated using established biochemical assays as previously described [22,23].

## Data Availability

The data supporting the findings of this study are included within the article. Additional data are available from the corresponding author.

## Abbreviations

AAALAC	Association for Assessment and Accreditation of Laboratory Animal Care International
AMD	Age-related macular degeneration
ANOVA	Analysis of variance
CCSEA	Committee for the Control and Supervision of Experiments on Animals
CpG	Cytosine–phosphate–guanine
DCFH-DA	2′,7′-Dichlorofluorescein diacetate
DNA	Deoxyribonucleic acid
ENU	N-ethyl-N-nitrosourea
FBS	Fetal bovine serum
Fe^2+^	Ferrous iron
GCL	Ganglion cell layer
H&E	Hematoxylin and eosin
HBSS	Hanks’ Balanced Salt Solution
IAEC	Institutional Animal Ethics Committee
IC_50_	Half-maximal inhibitory concentration
INL	Inner nuclear layer
JmjC	Jumonji C
KDM	Lysine (K) demethylase
NFL	Nerve fiber layer
ONL	Outer nuclear layer
OPL	Outer plexiform layer
OS	Outer segment
PCR	Polymerase chain reaction
PR	Photoreceptor layer
ROS	Reactive oxygen species
RPE	Retinal pigment epithelium
SD	Standard deviation
WST-1	Water-soluble tetrazolium salt-1
WT	Wild-type
